# Comparison of long-term kidney functions after radical nephrectomy and simple nephrectomy

**DOI:** 10.7717/peerj.6701

**Published:** 2019-04-12

**Authors:** Erkan Olcucuoglu, Senol Tonyali, Sedat Tastemur, Yusuf Kasap, Mehmet Emin Sirin, Eymen Gazel, Esin Olcucuoglu, Oner Odabas, Can Ates, Mahmut Taha Olcucu

**Affiliations:** 1 Clinic of Urology, University of Health Sciences, Turkiye Yuksek Ihtisas Training and Research Hospital, Ankara, Turkey; 2 Clinic of Urology, Kırsehir State Hospital, Kirsehir, Turkey; 3 Clinic of Urology, Acibadem University, Acibadem Ankara Hospital, Ankara, Turkey; 4 Clinic of Radiology, University of Health Sciences, Turkiye Yuksek Ihtisas Training and Research Hospital, Ankara, Turkey; 5 Department of Biostatistics, Yuzuncu Yil University, Van, Turkey; 6 Clinic of Urology, University of Health Sciences, Sultan Abdulhamit Han Training and Research Hospital, Istanbul, Turkey

**Keywords:** Nephrectomy, Chronic Kidney Disease, GFR, Renal carcinoma

## Abstract

**Objective:**

To determine if there is a difference in proceeding to CKD between patients who had undergone radical nephrectomy (RN) and simple nephrectomy (SN) for different indications by comparing the short- and long-term renal function.

**Materials and Methods:**

We retrospectively analyzed the records of all patients who underwent nephrectomy (either for malign or benign indications) in our clinic between January 2007 and September 2017. The patients were divided into 2 groups according the type of surgery: 1) Radical nephrectomy Group, 2) Simple Nephrectomy Group. Renal function was evaluated with Glomerular Filtration Rate (GFR) calculated using the MDRD formula.

**Results:**

A total of 276 patients were included in the study. There were 202 patients in RN Group and 74 patients in SN Group. The mean age of the patients in RN Group and SN Group were age 59,2 ± 11,5 and 49,9 ± 15,1 years, respectively (*p* = 0.001). GFR levels of patients in RN Group versus SN Group were as follows: Preoperative period: 84.9 vs. 81 mL/min/1.73 m^2^; postoperative 1st day: 60.5 vs. 84.4 mL/min/1.73 m^2^, postoperative 1st month 58.9 vs. 76 mL/min/1.73 m^2^, postoperative 1st year: 59.5 vs. 74.1 mL/min/1.73 m^2^; at last control 60.3 and 76.1 mL/min/1.73 m^2^. While preoperative GFR was found to be similar in two groups (*p* = 0.26), postoperative GFR values were found to be significantly lower in Group RN (*p* < 0.001). In comparison of the decrease in GFR in two groups at last follow-up, significantly higher decrease was observed in RN Group, 29% vs. 6%, (*p* < 0.05).

**Conclusion:**

The decrease in GFR exists more common and intensive after RN compared to SN. In long-term, compensation mechanisms that develop after sudden nephron loss like radical nephrectomy deteriorates kidney function more than gradual nephron loss as in benign etiologies which indicates simple nephrectomy.

## Introduction

Chronic Kidney Disease (CKD) is a common disease around the world and there is a steady increase in its prevalence. In developed countries, its prevalence is about 11% and is in inverse proportion with the socio-economic level (Webster et al., 2017). CKD is diagnosed as the estimated glomerular filtration rate (eGFR) < 60 mL/min/1.73 m^2^ or the urinary albumin/ creatinine (Cr) ratio (UACR) > 30 mg/g or evidence of kidney injury for more than 3 months, even in the presence of normal GFR (Webster et al., 2017; Levey et al., 2011). CKD presents with increase in blood Cr level by deteriorating the functions of the kidney. However, the level of Cr does not increase until the kidney parenchymal damage reaches to a certain level.

GFR reflects the function of kidney and described as the amount of blood filtered by the kidney in a minute. GFR is affected by age, sex, race (white and black) and blood Cr and, it is calculated by the formulation of these variables. Normal GFR level is regarded to be 125 mL/min/1.73 m^2^ and when it decreases to 60 mL/min/1.73 m^2^, it is considered to be CKD. CKD is divided into five subgroups according to GFR level; and, when GFR decrease under 15 mL/min/1.73 m^2^, end stage kidney failure occurs and renal replacement therapies are required (Webster et al., 2017; James, Hemmelgarn & Tonelli, 2010).

The absence of a kidney can be either congenital or acquired (such as surgery) and it is a risk factor for CKD (Schreuder, 2018; Chevalier, 2009). Nephrectomy is performed mainly for three indications: (1) Radical Nephrectomy (RN) performed for malignancies; (2) Simple Nephrectomy performed in case of kidney function loss due to benign factors like urinary stone disease, infection and trauma; (3) Donor Nephrectomy (DN) performed for kidney donation in renal transplantation. It is important to take care of preoperative and postoperative kidney functions in all these three surgeries. Regular examination is vital in preventing the progression to CKD in those patients by following the renal parenchymal changes. In case of CKD occurrence, it is a highly recommended to diagnose and control the diseases like hypertension (HT) and diabetes Mellitus (DM) that cause parenchymal damage. On the other hand, even if the primary disease is treated properly, CKD becomes inevitable when the damage in kidney parenchyma exceeds a certain point. This certain point can be explained as the decrease of GFR below 45 mL/min/1.73 m^2^ (Lane et al., 2015).

Currently, the impact of the type of nephrectomy (either radical or simple nephrectomy) on long-term renal function is not well established. In this study, we aimed to determine if there is a difference in proceeding to CKD between patients who had undergone radical and simple nephrectomy for different indications by comparing the short- and long-term renal function.

## Materials & Methods

After obtaining the approval of University of Health Sciences, Ankara Turkiye Yuksek Ihtisas Training and Research Hospital review board (IRB Approval Number: 28/12/2017-30-13504) we retrospectively analyzed the records of all patients who underwent nephrectomy (either for malign or benign indications) in our clinic between January 2007 and September 2017.

The examined parameters included patient’s demographics, preoperative and postoperative serum biochemical tests and urinalysis, radiological examination results and type of surgery. Inclusion criteria were as follow: (1) Preoperative GFR ≥ 60 mL/min/1.73 m^2^; (2) not having a history of proteinuria, urinary tract infection or other diseases like DM, HT and obesity; (3) No pathological finding (stone, mass, hydronephrosis) in contralateral kidney on radiological examination both in preoperative and postoperative episodes. Patients with a history of nephrotoxic drug use and patients whom all records and follow-up were not available were excluded from the study.

GFR was calculated by using the modification in diet and renal disease (MDRD) formula: [GFR = 186 × Serum creatine ^−1.154^ ×  age ^−0.203^ × 1.212 (if the patient is black) 0.742 (if the patient is female)]. Proteinuria was assessed as either negative or positive by using the dipstick technique.

RN patients follow-up visits were performed at postoperative 1st month, 3rd month, 6th month, 12th month, 18th month, 24th month then annually, including complete blood count, serum biochemical analysis, urinalysis, abdominal imaging and chest X-ray. SN patients did not have 3rd month control and chest X-ray during the follow-up.

### Statistical analysis

IBM SPSS package program version 20.0 (Armonk, NY, USA) was used for statistical analysis and statistical significance was set at *p* < 0.05. Shapiro Wilk was used to test the normality of distribution, and Levene’s test were employed to assess the homogeneity of variants. Student’s *T*-test and/or Mann Whitney *U* test was used in comparison of groups. The comparison of categorical variants was performed Chi square and Fisher’s exact tests. The variation between groups, changed according to time and group, and time interaction were analyzed with Recursive Identification ANOVA and Generalized Estimating Equations (GEE). Results were given with average ± standard error and 95% confidence intervals, as well as providing odds ratios and confidence intervals for GEE. Regression analysis was used to determine the predictors of progression to CKD.

## Results

The medical records of 446 patients who had undergone nephrectomy in our institution were reviewed. 170 patients with accompanying diseases or unavailable data were excluded. A total of 276 patients whom 167 were male and 109 were female, were included in the study. There were 202 patients in the RN Group and 74 patients in the SN Group. The ratio of male to female was 1.6 (126/76) in Group 1 and 1.2 (41/33) in Group 2 (*p* = 0.29). The mean age of the patients in the RN Group and the SN Group were age 59,2 ± 11,5 and 49,9 ± 15,1 years, respectively (*p* = 0.001). Follow-up times of the two groups were similar, 24.3 ± 23.1 versus 27.2 ± 27.2 months, (*p* = 0.41) (Table 1).

**Table 1:** Demographic characteristics and pathological classification of patients.

Variables	Radical nephrectomy (*n*, %)	Simple nephrectomy (*n*, %)	*p*
Sex			
Male	126, 62.4%	41, 55.4%	0.294
Female	76, 37.6%	33, 44.6%	
Age (Years, Mean ± SD)	59.2 ± 11.5	49.9 ± 15.1	<0.001
Monitoring time (Month, Mean ± SD)	24.3 ± 23.1	27.2 ± 27.2	0.413
Tumor stage			
T1a	51, 25.5%		
T1b	71, 35.5%		
T2a	36, 18.0%		
T2b	16, 8.0%		
T3a	22, 11.0%		
T4	4, 2.0%		
Pathological diagnosis			
clear cell	135, 67.5%		
Papillary Type 1	26, 8.0%		
Papillary Type 2	2, 1.0%		
Chromophobe	6, 3.0%		
Oncositoma	15, 7.5%		
Angiomyolipoma	4, 2.0%		
Transitional cell carcinoma	10, 5.0%		
Collecting duct carcinoma	2, 1.0%		
Sarcomatoid	5, 2.5%		
Hydatid cyst	3, 1.5%		
Neuroendocrine tumor	2, 1.0%		
Chronic Pyelonephrit		68, 91.9%	
Atrophic kidney		6, 8.1%	

GFR levels of patients in the RN Group versus SN Group were as follows: Preoperative period: 84.9 vs. 81 mL/min/1.73 m^2^; postoperative 1st day: 60.5 vs. 84.4 mL/min/1.73 m^2^, postoperative 1st month 58.9 vs. 76 mL/min/1.73 m^2^, postoperative 1st year: 59.5 vs. 74.1 mL/min/1.73 m^2^; at last control 60.3 and 76.1 mL/min/1.73 m^2^ (Table 2). Recursive Identification ANOVA model was employed to compare the GFR levels at aforementioned times between two groups; and while preoperative GFR was found to be similar in two groups (*p* = 0.26), postoperative GFR values were found to be significantly lower in the RN Group (*p* < 0.001).

**Table 2:** GFR levels of patients at pre- and post-operative period.

**Group**	**Radical Nephrectomy**			**Simple Nephrectomy**		
GFR value (mL/min/1.73m^2^)	**Mean**	**Minimum**	**Maximum**	**Mean**	**Minimum**	**Maximum**
Preoperative within 1 month	84.93	60	180	81.04	60	131
Postoperative 1st Day	60.54	27	127	84.49	41	156
Postop 1st Month	58.98	26	95	76.09	46	123
Postop 1st Year	59.50	32	99	74.19	36	104
Last control	60.35	23	116	76.17	19	106

**Figure 1:**
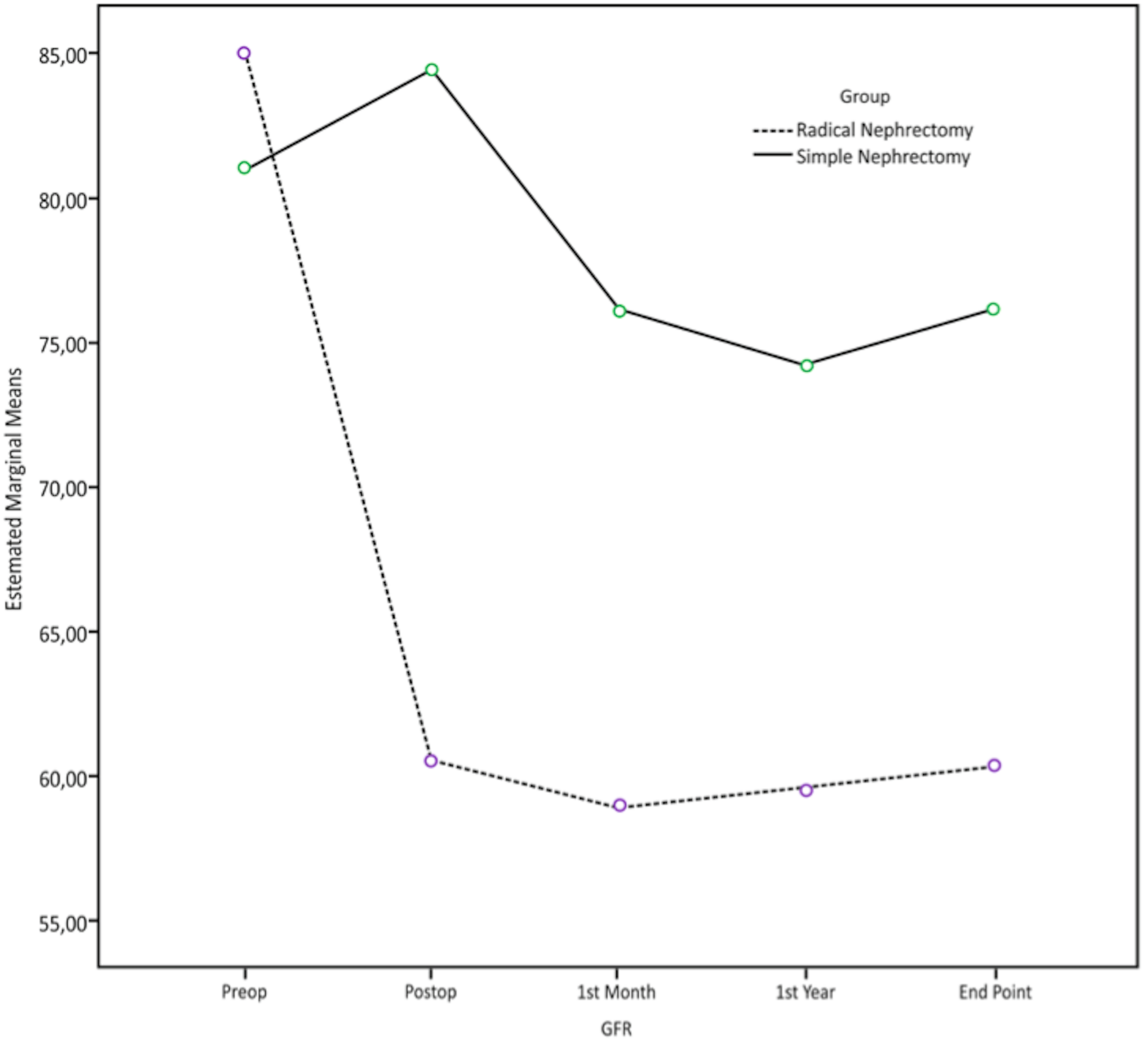
Alterations in GFR with time in patients who underwent radical nephrectomy or simple nephrectomy.

In comparison of the decrease in GFR in two groups at last follow-up, significantly higher decrease was observed in the RN Group, 29% vs. 6%, (*p* < 0.05) (Fig. 1).

According to GFR levels in last controls, while there is a development of CKD in 44.1% of patients in Group 1 developed CKD while this rate was 16.2% in Group 2. The type of the surgery, age at the time of surgery, GFR levels at given times (preoperative, postoperative; 1st day, 1st week, 1st year, last control time) were found to be significantly different between patients who developed CKD and who did not. No difference was observed regarding the sex and the follow-up time. (Table 3).

**Table 3:** Comparison of patients that developed and did not develop CKD.

Variables		End Point CKD NO (GFR > 60 ml/min/1.73m^2^) (*n*, %) (Mean ± SD)	End Point CKD YES (GFR < 60 ml/min/1,73m^2^) (*n*, %) (Mean ± SD)	*p*
Group Radical Neph		113, 55.9%	89, 44.1%	<0.001
Simple Neph.		62, 83.8%	12, 16.2%	
Sex	Male	99, 59.3%	68, 40.7%	0.078
	Female	76, 69.7%	33, 30.3%	
Age		52.7 ± 13.3	63.7 ± 3.5	<0.001
Preoperative GFR		91.7 ± 21.7	77.2 ± 11.7	<0.001
Postoperative GFR		76.3 ± 20.1	55.8 ± 14	<0.001
Postop 1. month GFR		72.1 ± 15.1	53.2 ± 10.5	<0.001
Postop 1. year GFR		71.5 ± 13.4	51.3 ± 10.9	<0.001
Last control GFR		75.9 ± 12.2	48.2 ± 8.3	<0.001
Follow-up (month)		24.5 ± 24.6	26.0 ± 23.6	0.610

In cox regression analysis older age and radical nephrectomy were found to be the significant predictors of proceeding to CKD [Exp (B): 1.04, *p* < 0.001 and Exp(B): 2.94, *p* = 0.001, respectively.]. Preoperative high GFR was found to be a negative predictor of proceeding to CKD [Exp(B):0.97, *p* < 0.001](Table 4).

**Table 4:** Cox regression analysis predicting proceeding to CKD.

**Variable**	**B**	**Exp (B)**	***P* value**
Age	0.41	1.04	<0.001
Preoperative GFR	−0.02	0.97	<0.001
Group (Radical vs. Simple Nephrectomy)	1.08	2.94	0.001
Gender Male	0.34	1.40	0.115

When we compare the periods (preoperative, postoperative; 1st day, 1st week, 1st year, last control time) regarding the development of CKD, while the variation between groups is significant (*p* < 0.001) and RN carries 6.5 times more risk than SN, the variation within the groups themselves is minor (*p* = 0.8) (Table 5). Comparison between groups that developed at risk of CKD). The group with time interaction is also found to be minor (*p* = 0.29). In particular, the insignificant variation in group with time interaction shows that groups tend to act similar regarding the assessments done on the set time points and that they follow the same pattern.

**Table 5:** Comparison between groups that are at risk of proceeding to CKD.

Factor	OR	St. Error	*p*	95% CI	
				Lower	Upper
Simple nephrectomy	**1**				
Radical nephrectomy	**6.500**	0.395	**<0.001**	2.99	14.11
**Postop**	1				
1st Month	1.228	0.179	0.253	0.863	1.747
1st Year	1.091	0.218	0.692	0.710	1.674
Last control	0.875	0.185	0.472	0.608	1.259
**RN × Postop**	1				
RN × 1st Month	0.888	0.436	0.785	0.377	2.088
RN × 1st Year	1.242	0.503	0.667	0.463	3.332
RN × Last control	1.597	0.382	0.221	0.755	3.379

## Discussion

As a result of nephron loss due to various reasons, in order to keep GFR in regular levels, other nephrons develop hyperfiltration and hyperplasia. This type of adaptation is known as compensatory renal hypertrophy (Kasiske et al., 1995; Brenner, Lawler & Mackenzie, 1996; Helal et al., 2012). Initially, it appears to be a useful method to keep GFR at a certain rate, however this causes particular structural changes like glomerulosclerosis and tubular atrophy. Clinically, these alterations represent as HT, proteinuria and decrease in GFR (Schreuder et al., 2008). HT and glomerulosclerosis triggers the existence of CKD by causing more nephron loss after a certain time (Schreuder et al., 2008).

In a study comparing the renal hemodynamic changes before and after donor nephrectomy, the authors reported that effective renal plasma flow increased by 40%, glomerular filtration rate increased by 59% and filtration fraction increased by 18%. However, any alteration in systolic and diastolic blood pressure was not observed. Also by reviewing 19 article related to renal functional changes after nephrectomy the authors concluded that GFR increases after unilateral nephrectomy by 17–80% and this increase was found to be positive correlated with time since nephrectomy (Guidi et al., 2008).

In the literature, strict follow-up of the patients with a solitary kidney is strongly recommended, since they have a high risk of progression to CKD (Webster et al., 2017; Schreuder, 2018; Chevalier, 2009). It is also recommended to make annual controls consisting of blood pressure measurement, serum Cr and GFR level and also radiological evaluation via renal ultrasonography (Schreuder, 2018). However, there is a lack of data about if there is a detectable difference at follow-up in patients with a solitary kidney according to the etiology of organ loss. Most of the studies in the literature were performed on pediatric patients with solitary kidneys, either by congenital or acquired. In their 9.1 years of study on children with congenital solitary kidney (CSK) (n: 44) and acquired solitary kidney (ASK) (n: 53), Abou Jaoudé et al. (2010) found higher GFR levels in CSK patients when compared to ASK patients [107.2 vs. 95.2 mL/min/1.73 m^2^, *p* < 0.01). While there was no patient with GFR <80 mL/min/1.73 m^2^ in CSK group, there were seven patients below this level in ASK group; and, all of those patients were observed to have had radical nephrectomy due to a renal mass. The authors concluded that the sudden increase of filtration of a kidney due to sudden contralateral kidney loss deteriorates the functions of the kidney in long-term. In other words, quick adaptation might indicate a negative impact on kidney functions in long term. In the KIMONO study, 4% of patients with CSK and 9% of patients with ASK were found to developed stage ≥3 CKD after 15 years of follow-up (Westland et al., 2013). In our study, we found that kidneys that develop adaptation gradually (SN group) preserve their functions better than kidneys that develop adaptation rather quickly (RN group). Moreover, we observed that 44.1% of patients with RN and 16.2% of patients with SN developed CKD; and regarding to the etiology of CKD, RN carries 6.5 times more risk than SN. While GFR level in RN group was higher than SN group during the preoperative period; in all postoperative periods, it was higher in SN group. In addition, the decrease rate in GFR was higher in patients with RN in postoperative period.

Most of the studies on the acquired absence of kidney in adults focus on the comparison of radical and donor nephrectomy. Thus, there is a scarce of data in comparison of radical nephrectomy and simple nephrectomy. RN is the standard treatment large renal masses. However, it causes a decrease in postoperative kidney functions and, eventually, brings along the risk of CKD occurrence. In the long-term period after RN, morbidity and mortality rate increase due to cardiovascular and cerebrovascular diseases emerging based on CKD (Zabell et al., 2018). As expressed in AUA and EUA guidelines, it is highly recommended to analyze the risk of postoperative CKD occurrence before performing RN and to apply partial nephrectomy without giving up on oncological principals (Ljungberg et al., 2017; Campbell et al., 2017).

There is no direct relation between RCC pathology and CKD however RCC patients are prone to progress to CKD due to surgical treatment. 25% of CKD patients are involved with CKD even before surgery. Nephron sparing treatment modalities must be considered to preserve renal function; on the other hand renal parenchyma outside the tumoral tissue might posses non-neoplastic diseases such as nephropathy and nephrosclerosis. These underlying pathologies might come apparent with surgical removal of functioning nephrons. Thus collaboration of urologists, pathologist and nephrologist is vital in management of renal cancer patients (Chang et al., 2014). In concordance with this suggestion, studies comparing the renal function after donor nephrectomy and radical nephrectomy reported better renal functions after donor nephrectomy (Gazel et al., 2015; Etafy et al., 2015). This might be a consequence of strict preoperative evaluation of renal donors.

After RN, GFR can decrease up to 35–40% in the postoperative first week. In order to compensate this decrease, in early period, contralateral kidney can develop sudden adaptation and hyperfiltration, along with an increase in GFR (Choi & Song, 2014; Chung et al., 2014). This compensation could be carry on during the five years follow-up period after RN (Chung et al., 2014). In their study, Sun et al. (2012) reported that five years after RN, 20.1% of the patients developed CKD. Similarly, Lane et al. (2013) found that 22% of the patients with normal preoperative GFR developed CKD after RN with an annual decrease of 0.7% in GFR. In the present study, we found that 44.1% of patients in RN group and 16.2% of patients in SN group developed CKD. In another study conducted at Memorial Sloan Kettering Cancer Center, it was found that 50% of patients who underwent RN returned back to their preoperative GFR levels. Furthermore, the GFR recovery of patients that have GFR <60 mL/min/1.73 m^2^ (though young and women) were better than those with GFR > 60 mL/min/1.73 m^2^ (Contrary to having HT) (Zabor et al., 2018). According to another study by Vergho et al. (2015), which compared RN and DN that leads nephron loss, the GFR decrease was found to be relatively low in RN group (32% vs. 34,2% *p* = 0.017). This decrease was considered as a sign of compensation of the contralateral kidney, which might have started in preoperative period of the patients, underwent RN. In their study, Gazel et al. (2015) reported 33.7% decrease in GFR”s of patients in RN group whereas 34.2% in DN group, after 20 months of follow-up (*p* = 0.783).

In the literature, having preoperative low GFR level (<60 mL/min/1.73 m^2^), old age, male gender, accompanying morbidities (such as DM, HT, obesity) are considered as risk factor for CKD occurrence after RN (Zabor et al., 2018). Among these factors, while preoperative GFR and the decrease rate in postoperative GFR are the most important predictors of the risk of postoperative CKD, preoperative GFR and age are the most important predictors of the mortality risk. In our study, RN, older age and preoperative low GFR were found to be the risk factors for CKD occurrence.

Simple nephrectomy (SN) is the removal of the kidney, after being nonfunctional, due to benign factors. In early postoperative period of SN, there is not a significant statistical change in GFR levels if the contralateral kidney is healthy, contrary to GFR decrease in RN. This might be due to the adaptation and compensatory mechanisms developed in preoperative period. In a study by Song et al. (2016), comparing SN and RN, mean preoperative GFR levels in SN and RN groups were 76.5 and 89.2 mL/min/1.73 m^2^ respectively; whereas 75.5 vs. 63.4 mL/min/1.73 m^2^ in the postoperative 7th day. At the end of the first year GFR of SN and RN patients were 72.6 and 65.9 mL/min/1.73 m^2^, respectively; and the higher decrease of GFR level in RN group was found to be statistically significant. In the same study, by assessing the kidney volume via computed tomography, it was concluded that the increase in kidney volume due to compensation was higher in SN group; and, it was clear that it has an impact on the protection of the postoperative renal functions. In another study comparing the decrease in GFR after RN, SN and DN, the decrease in GFR was highest after RN and lowest after DN. This was considered to be a result of the fact that individuals in DN group were younger and healthy, and they had no accompanying morbidity. After two years of follow-up, the difference in GFR was found to be significant between RN and SN, or RN and DN. At postoperative 6th years, while 41.6% of patients in RN group and 8.6% of patients in SN group developed stage 3 CKD, only 3 patients in DN group developed stage 1 CKD (Verma et al., 2016).

Age is one of the leading factors that negatively affect GFR levels. After a certain age, especially 40 years, the GFR decreases approximately 1% annually (Michels et al., 2010). This might be a consequence of histopathological changes that occur in the kidney parenchyma during aging process. In addition, it is necessary to know that at follow-up compensation will not be enough because particularly RN patients are relatively older thus GFR will continue to decrease. In our study, the difference in average age between the two groups has a noteworthy effect on pre- and post-operative GFR.

Finally, it has been shown that nephron loss in a kidney triggers compensatory mechanisms in the contralateral kidney. However, it is relatively less discovered in the literature how the pace of the compensatory process, sudden or gradual, affects the preservation of the remaining kidney’s function. In our study, we observed that at two years of follow-up kidney functions are preserved better and CKD occurred less in SN group.

Our study is also not without limitations. This is a retrospective study covering a long time span. This might resulted in selection bias and differences in ultrasonographic examinations due to different radiologist. Another issue, which must be discussed, is the older age of patients in RN group compared to SN group. However, preoperative GFR levels being similar in the two groups might overcome this confusing issue.

## Conclusions

The decrease in GFR exists more common and intensive after RN compared to SN. In the long term, compensation mechanisms that develop after sudden nephron loss like radical nephrectomy deteriorates kidney function more than gradual nephron loss as in benign etiologies which indicate simple nephrectomy.

##  Supplemental Information

10.7717/peerj.6701/supp-1 Data S1 Comparison of GFR after radical and simple nephrectomy

## Additional Information and Declarations

### Data Availability

The following information was supplied regarding data availability: The raw data is available as a Supplemental File.
